# Prenatal Tobacco and Alcohol Exposure and Cortical Change Among Youths

**DOI:** 10.1001/jamanetworkopen.2025.16729

**Published:** 2025-06-20

**Authors:** Andrew T. Marshall, Shana Adise, Eric C. Kan, Elizabeth R. Sowell

**Affiliations:** 1 Children’s Hospital Los Angeles, Los Angeles, California; 2 University of Southern California, Los Angeles

## Abstract

**Importance:**

The associations of prenatal alcohol exposure (PAE) and prenatal tobacco exposure (PTE) with adolescent neuroanatomical development are typically evaluated cross-sectionally. It is unclear whether observed effects persist throughout life or reflect different developmental trajectories.

**Objective:**

To examine whether PAE and PTE are associated with early-adolescent cortical structure and development.

**Design, Setting, and Participants:**

This cohort study included children aged 9 to 12 years who participated in the Adolescent Brain Cognitive Development (ABCD) Study’s first 2 neuroimaging time points (data collected 2016-2021) at 21 US study sites. Data analysis was conducted from March 2024 to March 2025.

**Exposures:**

PAE and PTE, based on caregiver reports of alcohol and tobacco use during pregnancy, both before and after pregnancy recognition.

**Main Outcomes and Measures:**

Cortical thickness (in millimeters) and cortical surface area (in millimeters squared) measured approximately 2 years apart, across 68 bilateral cortical regions. Summary scores from the Behavioral Inhibition and Behavioral Activation Scale, the Child Behavior Checklist, the Sleep Disturbances Scale for Children, and the Urgency, Perseverance, Premeditation, and Sensation Seeking Scale were collected.

**Results:**

At baseline data collection, the 5417 youth participants (2912 [53.8%] assigned male at birth; 724 [13.4%] Black, 1048 [19.3%] Hispanic, and 3640 [67.2%] White) had a mean (SD) age of 9.9 (0.6) years; the mean (SD) age at the second appointment was 11.9 (0.6) years. Cortical thickness decreased with age. Cortical surface area either expanded or contracted with age, depending on region. PAE was not associated with cortical structure (main correlation) or development (PAE × age interaction). PTE had false discovery rate–corrected main correlations with cortical thickness in the bilateral parahippocampal and left lateral orbitofrontal cortices (eg, right parahippocampal: |*r_p_*| = 0.04; *P* < .001) and was associated with faster rates of cortical thinning (PTE × age interactions) in medial and anterior frontal lobe regions (eg, right rostral middle frontal: |*r_p_*| = 0.04; *P* < .001). Post hoc analyses on PTE’s associations with cortical structure and development among children whose mother continued tobacco use after pregnancy recognition and among those whose mother did not also use alcohol had weaker effect sizes. Exploratory developmental-outcome analyses suggested that faster cortical thinning was associated with more externalizing behavior and sleep problems (eg, right pars orbitalis and externalizing behavior: |*r_p_*| = 0.04, *P* = .003), primarily in those with PTE.

**Conclusions and Relevance:**

In this study, PTE was correlated with cortical thickness development. Analyzing developmental trajectories informs not only how PTE and PAE affect cortical structure (and related behavioral outcomes) but also how the cortex develops long after prenatal exposures occurred. Future analyses involving cotinine biomarkers of PTE would enhance the temporal resolution of the ABCD’s PTE-related queries of tobacco use before and after learning of the pregnancy.

## Key Points

**Question:**

How does prenatal tobacco and/or alcohol exposure affect brain structure and longitudinal development in early adolescence?

**Findings:**

In this cohort study of 5417 youths aged 9 to 12 years, prenatal tobacco exposure was associated with faster rates of cortical thinning in the frontal lobe; prenatal alcohol exposure had no association with cortical structure and change. Exploratory developmental-outcome analyses suggested differential brain-behavior associations among youths with PTE.

**Meaning:**

These findings suggest that prenatal substance exposure affects not only cortical structure but also how cortical development long after those exposures occurred.

## Teaser

This cohort study evaluates whether prenatal tobacco and alcohol exposure are associated with cortical development among youth participants in the Adolescent Brain Cognitive Development study.

## Introduction

Environmental factors influence healthy development during early childhood and adolescence.^1^ For example, lead-exposure risk more detrimentally affected brain structure in youth in more socioeconomically disadvantaged families.^2^ Such neurotoxic effects are also associated with prenatal alcohol exposure (PAE) and prenatal tobacco exposure (PTE).^3^ Despite product labels advising otherwise,^4^ many pregnant individuals use alcohol or tobacco (either before or after knowing of the pregnancy).^5,6^ PAE is associated with smaller total and regional brain volumes and poorer connectivity.^7^ PTE is associated with reduced regional cortical thickness, surface area, and volume.^3,8,9,10,11,12,13,14,15^ While research has investigated PAE’s and PTE’s cross-sectional effects,^3,11^ less is known about neuroanatomical developmental trajectories given PTE and PAE long after they occur, which could elucidate, eg, whether individuals with PTE generally have thinner cortices or show faster rates of cortical thinning during childhood and adolescent development.

To address these questions, we analyzed data from the Adolescent Brain Cognitive Development (ABCD) Study, an ongoing, multisite longitudinal research study (eFigure 1 in Supplement 1).^16^ Past ABCD analyses showed that participants with PTE had smaller cortical surface areas, smaller cortical volumes,^12^ smaller subcortical volumes, and lower gray–white matter contrast^17^ at its first 2 neuroimaging time points (ie, early adolescence). Another ABCD study evaluated whether PAE’s and PTE’s associations with brain development and behavior were consistent between time points,^18^ but none of these studies specifically considered the change between assessments, which may clarify adolescent neuroanatomical developmental trajectories (and address ABCD’s central objectives^19^). Here, we examined whether PAE and PTE were associated with cortical thickness and surface area development across time points, hypothesizing (given past research) that PAE and PTE would be associated with greater cortical thinning (reduced and more rapidly decreasing thickness) and faster surface contraction (reduced and more rapidly decreasing area). Because early pregnancy exposures (eg, before pregnancy is known) may have weaker neuroanatomical and neurodevelopmental effects than later or continued exposure^9,20,21^ and dual PAE-PTE use may have unique effects beyond either alone,^11,22^ post hoc analyses examined whether significant PAE × age and PTE × age interactions were present in (1) children of individuals who continued using alcohol or tobacco after having learned of the pregnancy and (2) children of individuals who reported using tobacco but not alcohol (or alcohol but not tobacco). Exploratory analyses evaluated whether PAE- and PTE-associated outcomes for cortical structure and development were associated with various developmental outcomes.

## Methods

### Participants

ABCD enrolled 11 878 youths aged 9 and 10 years at 21 sites (eFigure 1 in Supplement 1) primarily using school-based enrollment.^23^ Our data (collected 2016-2021) came from 2021’s ABCD 4.0 data release,^24^ which included baseline and 2-year data for 11 876 and 10 414 youths, respectively (the 2 neuroimaging time points in this release). Centralized institutional review board (IRB) approval was obtained from UC San Diego. Study sites obtained approval from their IRBs. Caregivers provided written informed consent and permission; youth provided written assent. Data collection and analyses complied with all ethical regulations. Our report was structured in accordance with Strengthening the Reporting of Observational Studies in Epidemiology (STROBE) guidelines.^25^

### Prenatal Exposure, Birth Weight, and Demographic Characteristics

At baseline, caregivers reported biological mothers’ substance use (1) before knowing of pregnancy but when they could have been pregnant with the youth participant and (2) after knowing of the pregnancy (response options: “Yes,” “No,” “Don’t know” [DK]). We focused on alcohol and tobacco use. Caregivers also reported youth’s birth weight.

Sex assigned at birth was included in all publicly downloadable data tables. Youth race and ethnicity were reported by each youth’s caregivers. These data were collected to ensure a demographically diverse sample in line with ABCD’s targets per the American Community Survey.^23^ For race, caregivers were allowed to select from the following non-mutually-exclusive options: Alaska Native, American Indian or Native American, Asian Indian, Black, Chinese, Filipino, Guamanian, Japanese, Korean, Native Hawaiian, other Asian, other Pacific Islander, Samoan, Vietnamese, White, and other race, with additional response options being “Refuse to Answer” and DK. Neither race nor ethnicity were included in statistical analysis, but for the sake of reporting the characteristics of the sample, these 18 response options were collapsed into 8 categories: American Indian or Alaska Native (those who selected Alaska Native or American Indian or Native American), Asian (those who selected Asian Indian, Chinese, Filipino, Japanese, Korean, Vietnamese or other Asian), Black, Native Hawaiian or Other Pacific Islander (those who selected Guamanian, Native Hawaiian, other Pacific Islander, or Samoan), other (those who selected other race or more than 1 race), White, and missing or undefined. For ethnicity, caregivers were asked whether they considered their child Hispanic, Latino, or Latino, with response options being “Yes,” “No,” “Refuse to Answer,” and DK.

### Neuroimaging

ABCD neuroimaging acquisition parameters, data collection, and processing procedures have been thoroughly described.^26^ We analyzed the thickness (in millimeters) and surface area (in millimeters squared) of 68 bilateral cortical regions (derived using FreeSurfer version 7.1.1 Desikan-Killiany atlas on acquired T_1_-weighted structural magnetic resonance imaging [sMRI] volumes).^27,28^ Whole-brain volume, intracranial volume, mean cortical thickness, and total surface area were also analyzed.

### Caregiver and Youth Self-Reports of Developmental Outcomes

Four caregiver and youth self-reports of youth developmental outcomes were analyzed: the Behavioral Inhibition and Behavioral Activation Scale (BIS-BAS) (self-report), the Child Behavior Checklist (CBCL) (caregiver report), the Sleep Disturbances Scale for Children (SDSC) (caregiver report), and the Urgency, Perseverance, Premeditation, and Sensation Seeking Scale (UPPS) (self-report).^29,30^ The eAppendix in Supplement 1 provides additional details.

### Statistical Analysis

#### Cortical Development

The eAppendix and eTable 61 in Supplement 1 outline participant inclusion and exclusion criteria for analyses. Participants were categorized into PAE and PTE groups (Table). Non-PAE and non-PTE designations meant caregivers reported not using alcohol or tobacco products before or after learning or knowing of the pregnancy; PAE and PTE designations meant caregivers did report such use. Given a priori plans to conduct post hoc analyses on PAE and PTE after learning or knowing of the pregnancy, we excluded participants whose caregivers were unaware of the pregnant person’s alcohol and tobacco use during that period, regardless of what they reported before learning or knowing of the pregnancy, leaving 5417 participants for analyses (1500 with PAE, 3917 with non-PAE, 739 with PTE, 4678 with non-PTE). We used χ^2^ tests to compare demographic distributions of the full ABCD cohort vs the sample here and between PAE vs non-PAE and PTE vs non-PTE groups.

**Table.** PAE and PTE Group Criteria Based on Caregivers’ Responses

Group	Knowing of pregnancy		Participants in subgroup (participants in analysis), No.^a^^,^^b^	Total participants in group (participants in analysis), No.^b^
	Before	After		
**PAE**				
Non-PAE	No	No	3925 (3917)	3925 (3917)
PAE	No	Yes	NR (NR)	1516 (1500)
	Yes	No	1345 (1338)	
	Yes	Yes	146 (138)	
	DK	Yes	NR (NR)	
Excluded from analysis, No.	No	DK	NR	272
	Yes	DK	NR	
	DK	No	207	
	DK	DK	52	
	Undefined	Undefined	NR	
**PTE**				
Non-PTE	No	No	4850 (4678)	4850 (4678)
PTE	No	Yes	NR (NR)	772 (739)
	Yes	No	493 (477)	
	Yes	Yes	272 (256)	
	DK	Yes	NR (NR)	
Excluded from analysis, No.	No	DK	NR	91
	Yes	DK	NR	
	DK	No	30	
	DK	DK	45	
	Undefined	Undefined	NR	

Abbreviations: DK, do not know; NR, not reported; PAE, prenatal alcohol exposure; PTE, prenatal tobacco exposure.

^a^ Values of small cell sizes (n < 20) were suppressed (ie, noted by NR).

^b^ The numbers in parentheses refer to the participants within each group who went into data analyses, given the participants that were excluded due to responses to the other prenatal exposure questions.

Linear mixed-effects models were performed on cortical thickness and surface area for 68 regions. The model for thickness was:

Thickness ~ Sex at Birth × Age^2^ + PAE × PTE × Age^2^ + Birth Weight + Whole Brain Volume + (1|MRI Device) + (1|Participant),

and the model for surface area was:

Surface-area ~ Sex at Birth × Age^2^ + PAE × PTE × Age^2^ + Birth Weight + Total Surface Area + (1|MRI Device) + (1|Participant).

Given PAE- and PTE-associated reductions in brain size,^9,31^ we controlled for participants’ whole-brain volume (WBV) and total surface area (TSA) when analyzing regional thickness and surface area, respectively (to not conflate differences in change due to differences in absolute size). The eAppendix in Supplement 1 provides details on the use and selection of WBV and TSA for analyses and the coding and treatment of categorical and continuous factors in the models. Sensitivity analyses including socioeconomic variables (household income, caregiver education) in these models did not substantially change interpretation of our results. Data were excluded as outliers if they exceeded more than 3 scaled median absolute deviations from the median (eAppendix in Supplement 1).^32^

Effect sizes for age are represented by partial correlation coefficients (*r_p_*), which account for all other variables.^33^ The strengths of modeled interactions and categorical factors were calculated similarly. The Benjamini-Hochberg^34^ false discovery rate (FDR) algorithm was used to correct for multiple comparisons. eTables 1-52 in Supplement 2 show model output for thickness and surface area models. The main text describes results that passed FDR correction; the eAppendix in Supplement 1 has additional results.

#### Exploratory Analyses: Developmental Outcomes

For regions exhibiting significant PTE × age or PAE × age interactions, we evaluated how developmental outcomes (BIS-BAS, CBCL, SDSC, UPPS) were associated with individual differences in age-associated structural change (annual percentage change [APC]):
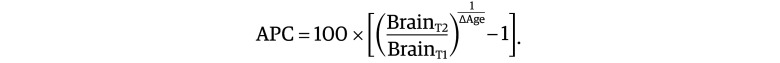
Brain_T2_ and Brain_T1_ were sMRI cortical metrics at the baseline (T1) and 2-year (T2) appointments, respectively; ΔAge, the T1-to-T2 change in age (in years). Two-sample *t* tests compared APC distributions between groups to corroborate results from mixed-effects models (PAE vs non-PAE; PTE vs non-PTE). For each region-outcome pair, we performed the following linear model:

Outcome ~ Sex + PAE (or PTE) + PTE (or PAE) × APC.

Outcome refers to BIS-BAS, CBCL, SDSC, or UPPS outcomes at the 2-year appointment or the difference score between those data from the baseline and 2-year appointments. Regional APC and developmental outcome data were excluded as outliers if they exceeded more than 3 scaled median absolute deviations from the median. Because these analyses were exploratory, we did not perform FDR correction (to fully explore potential associations). The eAppendix in Supplement 1 presents additional details.

Statistical analyses were performed in MATLAB R2022b version 9.13 (The MathWorks). The statistical significance level was set at .05 unless otherwise specified.

## Results

### Sample Characteristics

At the baseline appointment, the 5417 youth participants (2912 [53.8%] assigned male at birth; 724 [13.4%] Black, 1048 [19.3%] Hispanic, and 3640 [67.2%] White) had a mean (SD) age of 9.9 (0.6) years; at the 2-year appointment, their mean (SD) age was 11.9 (0.6) years. Compared with the full ABCD cohort, this sample’s youths were more likely to have male sex assigned at birth, live in socioeconomically advantaged families, and be reported by their caregivers as White and not Hispanic or Latino/a/x (eTable 62 in Supplement 1). Those with PAE (vs those without) and those without PTE (vs those with) were more likely to live in socioeconomically advantaged families (eTables 63 and 64 in Supplement 1).

### Prenatal Exposure

#### Age

**Figure 1.**
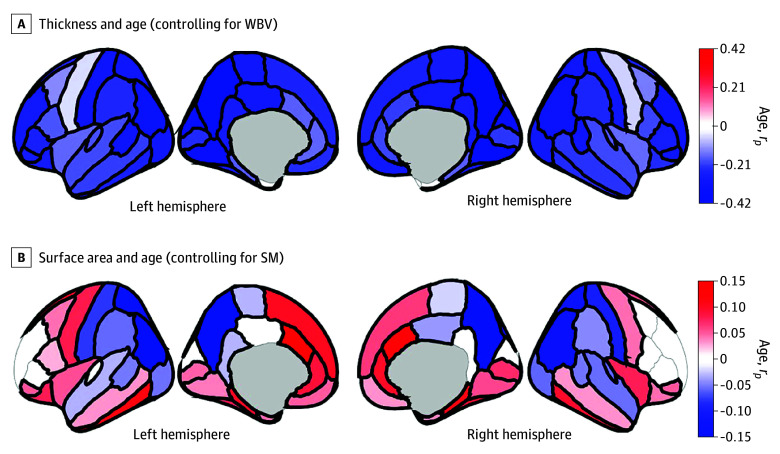
Associations Between Participant Age and Brain Structure Regions are color coded with respect to the color bars on the immediate right of each panel. (Note the different scales of the color bars.) Red- or blue-shaded regions indicate that those associations had *P* values less than .05; regions that have thick borders indicate that those associations also passed false-discovery rate correction. These images were generated in MATLAB using data from the ggseg toolbox in R.^35^ SM indicates total surface area; WBV, whole-brain volume.

There were FDR-corrected age-associated decreases in thickness in 65 regions (Figure 1A).^35^ For surface area, 56 regions showed FDR-corrected age-associated changes, with 34 expanding (eg, posterior-frontal, superior-temporal regions) and 22 contracting (ie, parietal, occipital, inferior-temporal regions) (Figure 1B).

#### PAE, PTE, and PAE × PTE Interactions

**Figure 2.**
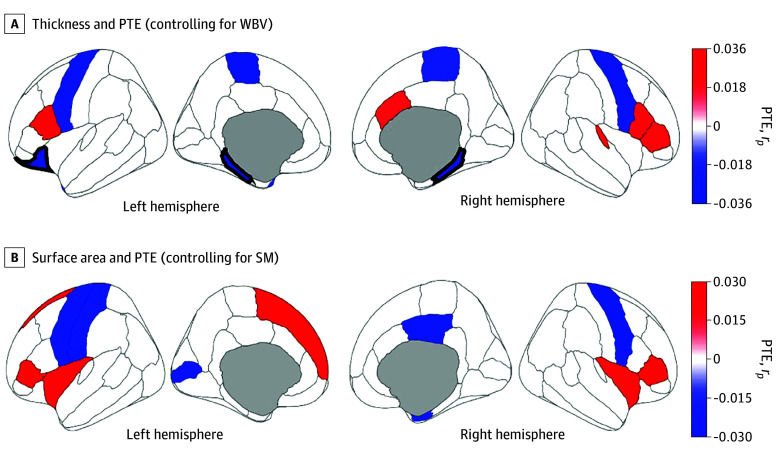
Associations Between Prenatal Tobacco Exposure (PTE) and Brain Structure Regions are color coded with respect to the color bars on the immediate right of each panel. (Note the different scales of the color bars.) Red- or blue-shaded regions indicate that those associations had *P* values less than .05; regions that have thick borders indicate that those associations also passed false-discovery rate correction. Blue-shaded regions reflect thinner cortices (A) or smaller surface areas (B) in those with PTE. Red-shaded regions reflect thicker cortices (A) or larger surface areas (B) in those with PTE. These images were generated in MATLAB using data from the ggseg toolbox in R.^35^ SM indicates total surface area; WBV, whole-brain volume.

There were no FDR-corrected associations of PAE or PAE × age interactions with cortical structure. Those with PTE had thinner cortices in the bilateral parahippocampal and left lateral orbitofrontal cortices (eg, right parahippocampal: |*r_p_*| = 0.04; *P* < .001) (Figure 2A; eTable 17 in Supplement 2). There were no FDR-corrected associations of PTE with surface area (Figure 2B).

**Figure 3.**
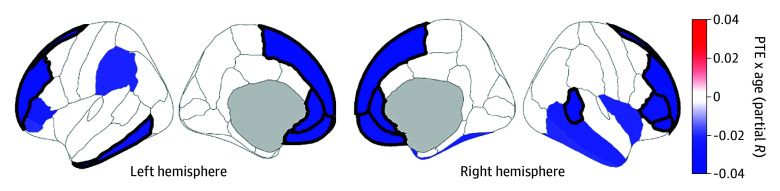
Prenatal Tobacco Exposure (PTE) × Age Interactions and Cortical Thickness Regions are color coded with respect to the color bar. Shaded regions indicate interactions that had *P* values less than .05; regions that have thick borders indicate that those interactions also passed false-discovery rate correction. The degree of shading indicates greater negative (or lesser positive) associations with age in individuals with PTE. Models controlled for whole brain volume. These images were generated in MATLAB using data from the ggseg toolbox in R.^35^

PTE was associated with faster cortical thinning (PTE × age) in 11 frontal (bilateral rostral middle frontal, bilateral superior frontal, bilateral medial orbitofrontal, bilateral rostral anterior cingulate, right pars orbitalis, left frontal pole, and right pars triangularis) and 2 temporal (right banks of the superior temporal sulcus and left inferior temporal) regions (eg, right rostral middle frontal: |*r_p_*| = 0.04; *P* < .001) (Figure 3; eTable 19 in Supplement 1). There were no FDR-corrected PTE × age interactions on surface area. There were no FDR-corrected PAE × PTE or PAE × PTE × age interactions for thickness (eTables 20-21 in Supplement 2) or surface area (eTables 27-28 in Supplement 2).

### Post Hoc Analyses

Post hoc analyses probed the associations of PTE with cortical thickness or thinning after learning of the pregnancy and the associations of PTE with cortical thickness or thinning when excluding individuals with PAE. For the knowing-of-pregnancy PTE post hoc analyses, there were no FDR-corrected main associations of PTE, PTE × age, PAE × PTE, or PAE × PTE × age with cortical thickness (eTables 45 and 47-49 in Supplement 2). For the PTE without PAE post hoc analyses, PTE was associated with (1) thinner right parahippocampal cortices and (2) faster thinning (PTE × age) of left frontal pole cortices (eg, left frontal pole cortex: |*r_p_*| = 0.04; *P* = .001) (eTables 51-52 in Supplement 2).

### Exploratory Analyses: Developmental Outcomes

**Figure 4.**
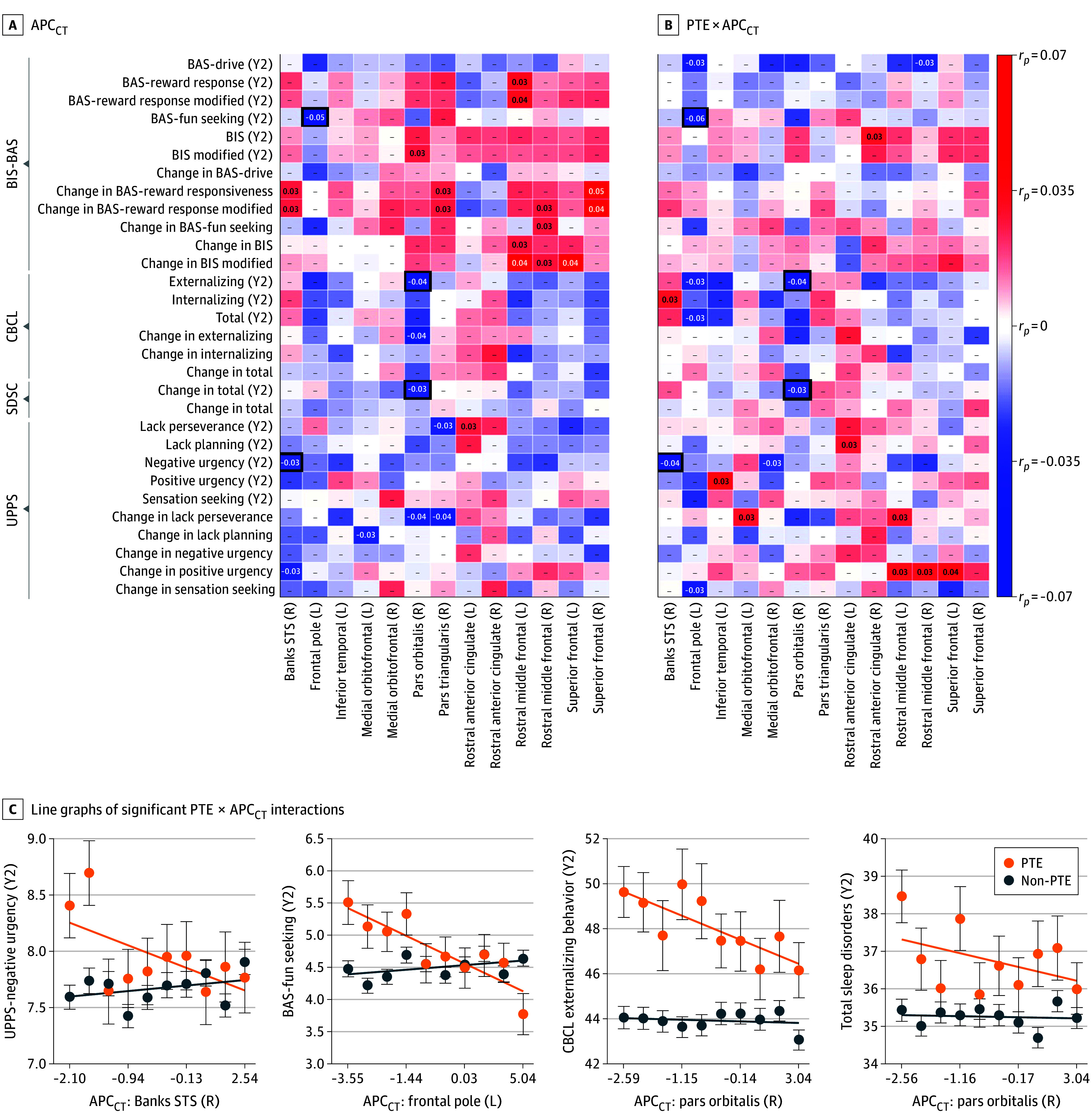
Exploratory Analyses Between Cortical-Thickness Development and Developmental Outcomes A, For the regions exhibiting significant prenatal tobacco exposure (PTE) × age interactions on cortical thickness (Figure 3), effect sizes (ie, partial correlation coefficients [*r_p_*]) for associations between annual percentage change for cortical thickness (APC_CT_) and developmental outcomes (left) and PTE × APC_CT_ interactions on developmental outcomes (right), controlling for prenatal alcohol exposure and sex at birth. Note that greater values of APC_CT_ reflect slower cortical thinning (or faster cortical thickening) and lower values of APC_CT_ reflect faster cortical thinning (or slower cortical thickening). The effect-size (*r_p_*) values are shown when the result was statistically significant (*P* < .05), but, for all cells, regardless of statistical significance, the degree of shading reflects the effect size (per the color bar). The thick boxes around 4 individual cells indicate the region-outcome pairs with both significant APC_CT_ associations and PTE × APC_CT_ interactions. Here, positive associations indicate that greater values of the developmental outcomes (or greater difference scores) were associated with slower cortical thinning (or faster cortical thickening), and negative associations indicate that greater values of these developmental outcomes (or greater difference scores) were associated with faster cortical thinning (or slower cortical thickening). B, Line graphs of significant PTE × APC_CT_ interactions for the 4 bordered cells in panel A. For ease of visualization, APC_CT_ data were partitioned into deciles with respect to the individuals in those analyses. Each data point is the mean ±1 standard error of the mean. The thick lines are simple regression lines for the data to convey direction. Banks STS indicates banks of the superior temporal sulcus; BIS/BAS, Behavioral Inhibition and Behavioral Activation Scale; CBCL, Child Behavior Checklist; L, left hemisphere; R, right hemisphere; SDSC, Sleep Disturbances Scale for Children; UPPS, Urgency, Perseverance, Premeditation, and Sensation Seeking Scale; and Y2, year 2.

For the 13 regions in which PTE was associated with faster cortical thinning, eFigure 2 in Supplement 1 shows APC for cortical thickness probability-density histograms for PTE and non-PTE participants. Figure 4A shows the effect sizes (*r_p_*) for associations of APC for cortical thickness with development outcome data as well as PTE × APC for cortical thickness interactions for these data (ie, BIS-BAS, CBCL, SDSC, and UPPS scores). There were both significant APC for cortical thickness associations and PTE × APC for cortical thickness interactions for 4 region-outcome pairs (Figure 4B; eTables 53-60 in Supplement 2). Faster regional cortical thickness (ie, lower APC for cortical thickness) was associated with greater or more negative urgency (UPPS: |*r_p_*| = 0.03; *P* = .045), fun-seeking (BIS-BAS: |*r_p_*| = 0.05; *P* = .001), externalizing behaviors (CBCL: |*r_p_*| = 0.04; *P* = .003), and sleep disorders (SDSC: |*r_p_*| = 0.03; *P* = .03) at the 2-year appointment, but these inverse associations were primarily driven by those with PTE (eg, right pars orbitalis and externalizing behavior, |*r_p_*| = 0.04; P = .01).

## Discussion

In this study, PTE’s longitudinal associations on cortical structure and development were stronger and regionally broader than PAE’s, with PTE associated with faster rates of frontal lobe cortical thinning (Figure 3). In post hoc analyses, these effect sizes were smaller, possibly due to systematic underreporting of PTE, differences in developmental timing, or loss of statistical power. Exploratory analyses suggested that cortical thinning rates were associated with developmental outcomes related to negative urgency, fun-seeking, externalizing behavior, and sleep disorders.

We know of 2 ABCD reports on PTE’s and PAE’s associations with cortical structure at multiple time points, both of which analyzed neuroimaging time points separately.^12,18^ Puga et al^12^ observed reduced entorhinal, precentral, and postcentral area given PTE, comparable with our results (albeit non–FDR-corrected here) (Figure 2B; eTable 24 in Supplement 2). Like Gu et al,^18^ we showed FDR-corrected thinner bilateral parahippocampal cortices in those with PTE (Figure 2A). While Gu et al^18^ reported significantly thicker right pars triangularis cortices (eTable 17 in Supplement 2), our results revealed significant PTE × age interactions there and in surrounding regions (eTable 19 in Supplement 2). Thus, by incorporating longitudinal trajectories, we portrayed broader patterns of faster frontal lobe cortical thinning given PTE (Figure 3). As noted, PTE is associated with reduced cortical thickness, surface area, and volume across regions,^3,8,9,10,11,12,13,14,15^ which was partially corroborated here (ie, some cortical regions were thicker and more expansive given PTE, albeit non–FDR-corrected) (Figure 2). Overall, our analyzing developmental trajectories of thickness and surface area informed how PTE and PAE may impact adolescent neuroanatomical development longitudinally, well after such prenatal insults occurred, more specifically addressing ABCD’s central objectives.^19^

PAE’s relatively negligible effects were contrary to our hypotheses. While 1 ABCD study similarly showed minimal PAE associations with cortical thickness, it showed broader PAE-associated increases in surface area.^18^ Another ABCD publication reported greater baseline regional cortical volumes and surface areas given PAE.^36^ Interestingly, the latter controlled for intracranial volume (ICV) in volumetric analyses. While it is unclear whether ICV was included in those surface area analyses, it is noteworthy that had our final analyses controlled for ICV instead of TSA, we also would have suggested that PAE’s associations with cortical area were widespread and significant (eTable 30 in Supplement 2), suggesting that covariate selection (which, here, was informed by developmental trajectories) may similarly affect understanding of variables of interest (eg, PAE, PTE).^37^

In contrast to PTE’s omnibus associations, there were smaller effect sizes in post-hoc analyses. These latter analyses included dramatically smaller sample sizes in the PTE group (omnibus analyses: n = 739; PTE after knowing of pregnancy analyses: n = 262; PTE with no PAE: n = 366), reducing analytical power. While these analyses could suggest that continued tobacco use after learning of the pregnancy restores or protects against tobacco’s neurotoxic effects from being used prior to learning of the pregnancy, it is likelier that tobacco use after learning of the pregnancy (and, presumably, before learning of it as well) was underreported, considering ABCD’s retrospective collection of these data approximately 10 years after pregnancy. While underreporting (via self-report) of tobacco use and smoking during pregnancy can be prevalent when measured prospectively,^38,39,40^ retrospective reports may be generally accurate and reliable,^41^ potentially reflecting reduced stigma in reporting such events when not pregnant,^42^ with there being good reliability between self-reports years apart.^43,44,45^ One study (which compared prospective identification of during-pregnancy smoking vs retrospective recall 14.5 years later) found that 9% of women (prospectively identified as smokers) retrospectively indicated not smoking during pregnancy (or having smoked but not after learning of the pregnancy).^41^ In another study comparing midwives’ records at first antenatal visits vs retrospective recall of during-pregnancy smoking approximately 11 years later, the highest proportion of discordant reports were in those whose records indicated they smoked cigarettes but retrospectively reported being nonsmokers.^42^ Thus, the weaker associations in our post hoc analysis of PTE after knowing of pregnancy (relative to the omnibus analyses) may be because those who did use tobacco after having learned of their pregnancy reported not doing so during ABCD’s retrospective data collection.

Alternatively, the omnibus interactions may have gone undetected in the post hoc analyses because such faster cortical thinning (within the omnibus analyses) had already occurred in those with PTE after knowing of pregnancy. In children aged 6 to 8 years, the largest effect sizes of PTE on cortical thickness were in those with PTE throughout the pregnancy, with no associations between PTE and cortical thickness when the pregnant person’s tobacco use ceased upon learning of the pregnancy^9^; in research of children aged 9 to 11 years showing a similar pattern of results as that study, the authors suggested that “smoking cessation as soon as pregnancy is known, if not before pregnancy, is not too late for offspring brain development.”^15^ However, PTE after knowing of the pregnancy may indeed be associated with differences in brain development, even if such effects take longer to manifest, as potentially observed here.

Given typical patterns of cortical thinning in adolescence^46^ (Figure 1), the differential rates of frontal cortical thinning given PTE (Figure 3) suggest 2 (not mutually exclusive) possibilities: cortical thinning is happening (1) faster or (2) earlier in those with PTE. In adolescence, reductions in cortical gray matter volumes (ie, products of cortical thickness and surface area) are presumably due to concurrent increases in white matter (ie, myelination) and/or decreases (ie, pruning) in synaptic and neuronal volume,^47,48^ with recent ABCD analyses suggesting the latter to be a better explanation of gray matter cortical volume loss.^49^ In aborted fetal tissue, PTE was associated with reduced neuronal count and altered DNA methylation (DNAm) in the dorsolateral prefrontal cortex.^50^ DNAm is associated with brain structure^51^ and may have a role in synaptic plasticity and pruning.^52^ PTE-associated DNAm differences persist into childhood^53^ and late adolescence.^54^ DNAm data are increasingly used to estimate epigenetic or biological age (vs chronological age),^55^ with accelerated epigenetic aging suggested to occur in current smokers (vs never or former smokers)^56^ and in those with prenatal exposure to tobacco smoke.^57^ Accelerated epigenetic aging is associated with thinner cortices in multiple brain regions in younger and older adults.^58,59^ Thus, mechanistically, PTE may not necessarily be associated with faster cortical thinning but earlier (potentially premature) cortical thinning (vs those with no PTE), due to PTE’s persisting effects on DNAm. This hypothesis could be further elucidated with longitudinal neuroimaging collection (which will be available in ABCD) plus biochemical DNAm analyses of whole-blood samples (which have been collected but not analyzed for such purposes in ABCD).

Exploratory analyses suggested that cortical thinning rates may have different implications for those with vs without PTE. PTE is associated with attention-deficit/hyperactivity disorder (ADHD),^60,61,62,63^ antisocial behavior,^64,65^ and schizophrenia.^66^ Past ABCD reports have identified PTE-associated increases in behavioral problems,^67,68,69^ body mass index, and sleep problems at baseline^68^ as well as caregiver-reported ADHD symptoms in youth.^70,71^ Less is known about relationships between cortical change and longitudinal outcomes (or changes in outcomes between time points). Here, in 4 region-outcome pairs with significant APC for cortical thickness associations and PTE × APC for cortical thickness interactions, inverse APC for cortical thickness–outcome associations were observed in those with PTE. In those without PTE, there were smaller (if any) associations, potentially suggesting that neurotoxicant exposure (even years afterwards) may predispose an exposed group to acute brain-behavior individual differences (ie, individual differences in behavior may be more subject to individual differences in brain structure given environmental insult); notably, we observed a similar result for lead-exposure risk, socioeconomic status, and cognitive performance.^2^ Future longitudinal ABCD investigations may help clarify whether brain-behavior associations are differentially malleable to environmental exposures or whether they also reflect earlier or faster changes in behavioral development.

### Limitations

This study has limitations. Our statistical models controlled for age, age squared, sex at birth, a whole-brain covariate, birth weight, and MRI device, omitting factors traditionally included in neuroimaging analyses (eg, race, socioeconomic status). However, sensitivity analyses including socioeconomic variables (but excluding participants with missing socioeconomic data) did not substantially change how we interpreted our results. This decision was primarily driven by (1) our specific hypotheses^72,73^ and (2) research identifying large demographic differences between caregivers of ABCD’s youth participants prenatally exposed vs not exposed to alcohol,^36,74^ tobacco,^10^ and/or cannabis^68^ (eTables 63 and 64 in Supplement 1)—such multicollinearity may attenuate or accentuate differences between exposed and nonexposed groups. While ABCD offers thousands of possible covariates to include in thousands of possible analyses, we acknowledge that our results should be treated as reflecting strengths of associations, not direct, causal pathways.

Given ABCD’s design, we cannot biochemically verify PTE or PAE (or lack thereof) and are limited by PTE and PAE being caregiver-reported years later. However, as ABCD collected participants’ deciduous teeth, there is the potential to extend our analyses to cotinine biomarkers of PTE^75^ to enhance the temporal resolution of ABCD’s PTE-related queries of tobacco use before and after learning of the pregnancy. Similarly, as ABCD began at ages 9 to 10 years, we cannot (in ABCD) evaluate PTE’s and PAE’s neuroanatomical effects before then, and we cannot yet evaluate how PAE and PTE impact later-adolescent cortical development. Furthermore, we did not evaluate whether PTE’s association with cortical development was present subcortically. But, as with potential cotinine measurements, ABCD provides a rich resource to understand how prenatal, postnatal, and early-childhood environments affect brain development.

## Conclusions

In this cohort study analysis of 5417 youths aged 9 to 12 years, we found that PTE was robustly associated with faster rates of frontal lobe cortical thinning during early adolescence, possibly due to such thinning happening at earlier ages in those with PTE. Given our investigation of early adolescent cortical change, we have provided key insight into how the prenatal environment may influence brain development through childhood into adolescence, long after neurotoxicant exposure.

## Notes

Supplement 1. **eAppendix.** Supplementary Methods
eReferences.
**eFigure 1.** US Map of the Locations of the Adolescent Brain Cognitive Development (ABCD) Study **eTable 61.** Inclusionary and Exclusionary Criteria for Analysis **eTable 62.** Participant Demographics **eTable 63.** Demographics of Youth Participants in This Study Reported by Caregivers as Being Prenatally Exposed and Not Exposed to Alcohol (PAE and Non-PAE) **eTable 64. **Demographics of Youth Participants in This Study Reported by Caregivers as Being Prenatally Exposed and Not Exposed to Tobacco (PTE and Non-PTE) **eFigure 2.** Annual Percentage Change (APC) for Cortical Thickness

Supplement 2. **eTable 1.** Thickness~Age(SM) **eTable 2.** Thickness~PAE(SM) **eTable 3. **Thickness~PTE(SM) **eTable 4.** Thickness~PAExAge(SM) **eTable 5.** Thickness~PTExAge(SM) **eTable 6.** Thickness~PAExPTE(SM) **eTable 7. **Thickness~PAExPTExAge(SM) **eTable 8.** Thickness~Age(ICV) **eTable 9.** Thickness~PAE(ICV) **eTable 10.** Thickness~PTE(ICV) **eTable 11.** Thickness~PAExAge(ICV) **eTable 12.** Thickness~PTExAge(ICV) **eTable 13.** Thickness~PAExPTE(ICV) **eTable 14.** Thickness~PAExPTExAge(ICV) **eTable 15.** Thickness~Age(WBV) **eTable 16.** Thickness~PAE(WBV) **eTable 17.** Thickness~PTE(WBV) **eTable 18.** Thickness~PAExAge(WBV) **eTable 19.** Thickness~PTExAge(WBV) **eTable 20.** Thickness~PAExPTE(WBV) **eTable 21.** Thickness~PAExPTExAge(WBV) **eTable 22.** Area~Age(SM) **eTable 23.** Area~PAE(SM) **eTable 24.** Area~PTE(SM) **eTable 25.** Area~PAExAge(SM) **eTable 26.** Area~PTExAge(SM) **eTable 27. **Area~PAExPTE(SM) **eTable 28.** Area~PAExPTExAge(SM) **eTable 29.** Area~Age(ICV) **eTable 30.** Area~PAE(ICV) **eTable 31.** Area~PTE(ICV) **eTable 32.** Area~PAExAge(ICV) **eTable 33.** Area~PTExAge(ICV) **eTable 34.** Area~PAExPTE(ICV) **eTable 35.** Area~PAExPTExAge(ICV) **eTable 36.** Area~Age(WBV) **eTable 37.** Area~PAE(WBV) **eTable 38.** Area~PTE(WBV) **eTable 39.** Area~PAExAge(WBV) **eTable 40.** Area~PTExAge(WBV) **eTable 41.** Area~PAExPTE(WBV) **eTable 42.** Area~PAExPTExAge(WBV) **eTable 43.** Thickness~Age(WBV) **eTable 44.** Thickness~PAE(WBV) **eTable 45.** Thickness~PTE(WBV) **eTable 46.** Thickness~PAExAge(WBV) **eTable 47.** Thickness~PTExAge(WBV) **eTable 48.** Thickness~PAExPTE(WBV) **eTable 49.** Thickness~PAExPTExAge(WBV) **eTable 50.** Thickness~Age(WBV) **eTable 51.** Thickness~PTE(WBV) **eTable 52.** Thickness~PTExAge(WBV) **eTable 53.** BISBAS~APC **eTable 54.** BISBAS~PTExAPC **eTable 55.** CBCL~APC **eTable 56.** CBCL~PTExAPC **eTable 57. **SDSC~APC **eTable 58.** SDSC~PTExAPC **eTable 59.** UPPS~APC **eTable 60.** UPPS~PTExAPC

Supplement 3.
Data Sharing Statement
