# Supplementary material for: Prenatal Tobacco and Alcohol Exposure and Cortical Change Among Youths
Source: JAMA Netw Open. 2025 Jun 20;8(6):e2516729. doi: 10.1001/jamanetworkopen.2025.16729 (PMC12181798; doi:10.1001/jamanetworkopen.2025.16729)
Supplement: Supplement 1. — eAppendix. Supplementary Methods eReferences. eFigure 1. US Map of the Locations of the Adolescent Brain Cognitive Development (ABCD) Study eTable 61. Inclusionary and Exclusionary Criteria for Analysis eTable 62. Participant Demographics eTable 63. Demographics of Youth Participants in This Study Reported by Caregivers as Being Prenatally Exposed and Not Exposed to Alcohol (PAE and Non-PAE) eTable 64. Demographics of Youth Participants in This Study Reported by Caregivers as Being Prenatally Exposed and Not Exposed to Tobacco (PTE and Non-PTE) eFigure 2. Annual Percentage Change (APC) for Cortical Thickness [file jamanetwopen-e2516729-s001.pdf]

Supplemental Online Content

Marshall AT, Adise S, Kan EC, Sowell ER. Cortical change among youths and prenatal tobacco and alcohol exposure. *JAMA Netw Open.* 2025;8(6):e2516729. doi:10.1001/jamanetworkopen.2025.16729

eAppendix. Supplementary Methods

eReferences.

eFigure 1. US Map of the Locations of the Adolescent Brain Cognitive Development (ABCD) Study

eTable 61. Inclusionary and Exclusionary Criteria for Analysis

eTable 62. Participant Demographics

eTable 63. Demographics of Youth Participants in This Study Reported by Caregivers as Being Prenatally Exposed and Not Exposed to Alcohol (PAE and Non-PAE)

eTable 64. Demographics of Youth Participants in This Study Reported by Caregivers as Being Prenatally Exposed and Not Exposed to Tobacco (PTE and Non-PTE)

eFigure 2. Annual Percentage Change (APC) for Cortical Thickness

This supplemental material has been provided by the authors to give readers additional information about their work.

© 2025 Marshall AT et al. *JAMA Network Open*.

eAppendix. Supplementary Methods

Inclusion/Exclusion Criteria

Per ABCD's neuroimaging data-release notes, 8 participants were excluded, as were 209 participants whose T_1w data were not recommended for analysis (remaining *n*=11,659). We removed 4,227 participants who did not have baseline and two-year sMRI data (remaining *n*=7,432) and 736 participants who had outlying/missing birthweight data (remaining *n*=6,696). (Birthweight was analyzed because PTE is associated with reduced birthweight[1] and smaller childhood/adolescent brain volume,[2] which are also associated with each other.[3]) Because ABCD includes siblings, we controlled for family relatedness by only including singletons or one sibling per family; we prioritized siblings based on PAE/PTE and data completeness (yes/no responses to PTE/PAE before/after learning of the pregnancy: PTE/Before, PTE/After, PAE/Before, PAE/After). If siblings had identical sets of yes/no/DK responses, we randomly selected one sibling from the corresponding family groups using MATLAB's *datasample* function (seed=1) [MATLAB Version: 9.13.0.2126072 (R2022b) Update 3]. Otherwise, we included the sibling based on (1) more "yes" responses to PTE/After and PAE/After pregnancy recognition, (2) more "yes" responses total, and (3) fewest DK responses (remaining *n*=5,713). See eFigure 2 for a graphical outline of the inclusion/exclusion process.

© 2025 Marshall AT et al. *JAMA Network Open*.

Caregiver and Self-Reports of Developmental Outcomes

The ABCD collection protocol for the following scales have been outlined previously.[4,5]

Behavioral Inhibition and Behavioral Activation Scale (BIS-BAS). The BIS-BAS, a 20-item scale on constructs related to sensitivities and behavioral responses to rewarding and aversive stimuli,[6] was self-completed by youth participants at the baseline and two-year appointments. Summary scores [for behavioral inhibition (BIS), behavioral activation (BAS) reward responsiveness, BAS-drive, and BAS-fun seeking] and modified summary scores (for BIS and BAS-reward responsiveness) were computed as described previously.[6,7]

Child Behavior Checklist (CBCL). The CBCL is a 113-item caregiver-completed questionnaire on their youth's behavioral problems from the past 6 months[8]; the CBCL was completed at the baseline and two-year appointments.[4,5] Summary scores were computed (and standardized) for internalizing-behavior, externalizing-behavior, and total problems.

Sleep Disturbances Scale for Children (SDSC). Caregivers completed the 26-item SDSC at the baseline and two-year appointments, assessing youth's sleep-related behaviors in the previous 6 months.[4,9] The total sleep-disorders score is a sum of scores of 6 subscales: (1) disorders of initiating and maintaining sleep, (2) sleep breathing disorders, (3) disorders of arousal/nightmares, (4) sleep-wake transition disorders, (5) disorders of excessive somnolence, and (6) sleep hyperhidrosis.

Urgency, Perseverance, Premeditation, and Sensation Seeking Scale (UPPS). A modified, 20-item UPPS scale was self-completed by youth participants at the baseline and two-year appointments.[4] The UPPS consisted of five subscales (4 items each) pertaining to (1) lack of perseverance, (2) lack of planning, (3) negative urgency, (4) positive urgency, and (5) sensation seeking.

Analysis. For each region in which there was either a significant PTE×Age or PAE×Age interaction, exploratory analyses were performed to evaluate if cortical developmental within those regions was associated with either (1) baseline-to-two-year-appointment changes in these developmental-outcome (behavioral/mental-health) data or (2) the two-year data. Simple difference scores were computed as $Behavior_{T2} – Behavior_{T1}$, in which T1 was the baseline appointment and T2 was the two-year appointment. The two-year data for BAS-drive (BIS-BAS), total sleep disorders (SDSC), lack-of-perseverance, lack-of-planning, and positive urgency (UPPS) were square-root-transformed to address positive skewness. Two-sample t-tests were performed to compare annual percentage change (APC) distributions between exposure groups to further verify results from the linear mixed-effects models (i.e., PAE vs. Non-PAE; PTE vs. Non-PTE). For each region with a significant PTE×Age or PAE×Age interaction and for each developmental two-year outcome or difference score, we performed linear models of the following form:

$$Outcome \sim Sex\text{-}at\text{-}Birth + PAE\ (or\ PTE) + PTE\ (or\ PAE) * APC$$

Sex-at-birth, PAE, and PTE were effect-coded categorical factors with male/female, Non-PAE/PAE, and Non-PTE/PTE, respectively, coded as -1/+1. APC was not transformed. As in the linear mixed-effects analyses of cortical development, effect sizes for APC are represented by partial correlation coefficients (r_p), which account for all other predictors.[10] For consistency, the strengths of PTE×APC or PAE×APC interactions were calculated similarly. The 95% CIs of effect sizes were derived from the sample variance of the partial correlation.[11] Due to the exploratory nature of these analyses, we did not perform false-discovery-rate (FDR) correction so as to fully explore potential associations between cortical change and neurodevelopment.

Whole-Brain Covariate Selection

Analysis. Past research has suggested PAE- and PTE-related reductions in total brain size.[2,12] Thus, to not conflate differences in change due to differences in absolute size, we first determined a whole-brain covariate for analyzing longitudinal change in regional thickness and surface area.[13] Due to their common usages as whole-brain covariates in ABCD,[13] we compared whole-brain volume (WBV), intracranial volume (ICV), and a summary metric (SM; either mean cortical thickness or total cortical surface area). Thus, for both cortical thickness and surface area, across 68 bilateral regions, we ran 4 linear mixed-effects models:

Age-Only:	$Brain \sim Age^2 + (1 \vert MRI\ Device) + (1 \vert Participant)$
Intracranial Volume (ICV):	$Brain \sim Sex * Age^2 + PAE * PTE * Age^2 + Birthweight + ICV + (1 \vert MRI\ Device) + (1 \vert Participant)$
Whole-Brain Volume (WBV):	$Brain \sim Sex * Age^2 + PAE * PTE * Age^2 + Birthweight + WBV + (1 \vert MRI\ Device) + (1 \vert Participant)$
Summary Metric (SM):	$Brain \sim Sex * Age^2 + PAE * PTE * Age^2 + Birthweight + SM + (1 \vert MRI\ Device) + (1 \vert Participant)$

Age (months), birthweight (total ounces), ICV, WBV, and SM (mean thickness or total area) were continuous, mean-centered predictors. While we focused on linear interactions, Age^2 (which inherently includes the lower-order linear term Age) (Wilkinson & Rogers, 1973) was included in all models given curvilinear changes in brain structure over longer stretches of adolescence.[14] Sex-at-birth, PAE, and PTE were effect-coded categorical factors with male/female, Non-PAE/PAE, and Non-PTE/PTE, respectively, coded as -1/+1. By-MRI-device and by-participant intercepts were random effects. All models used a random initial value for iterative optimization. For each region-by-region analysis, participants' data were considered

© 2025 Marshall AT et al. *JAMA Network Open*.

outliers (and excluded) if either of their datapoints exceeded more than three scaled median absolute deviations away from the median.[15] This resulted in between 10 and 241 participants excluded from thickness analyses (*n*'s=5,176-5,407); from surface-area analyses, between 4 and 107 participants (*n*'s=5,310-5,413).

Separately, for thickness and surface area, the more optimal whole-brain covariate was selected based on two criteria: (1) a Pearson's-*r* correlation between the fixed-effects coefficients (across 68 ROIs) from the age-only models and those from the ICV, WBV, and SM models; higher correlations reflected greater similarities between two sets of coefficients; (2) the residual total sum-of-squares (TSS) between each of the ICV, WBV, and SM models and the age-only model (sum of the squared differences between the coefficients from each of the former and the latter); smaller values reflected greater similarities between sets of coefficients. The chosen set of models were used to evaluate PTE×Age/PAE×Age interactions on brain structure.

Effect sizes are represented by partial correlation coefficients (r_p), which account for all other predictors.[10] The strengths of modelled interactions and categorical factors were calculated similarly. The 95% CIs of effect sizes were derived from the sample variance of the partial correlation.[11]

Results. The linear coefficients for age from age-only cortical-thickness models were more comparable to those from the WBV and ICV models [WBV: $r>.99$, $p<.001$, TSS=1.14×10^{-6}; ICV: $r>.99$, $p<.001$, TSS=1.25×10^{-6}] than models controlling for mean thickness ($r=.89$, $p<.001$, TSS=1.11×10^{-4}]. Accordingly, we controlled for WBV in cortical-thickness models. In surface-area models, we controlled for total area (SM: $r>.99$, $p<.001$, TSS=0.72; WBV: $r=.96$, $p<.001$, TSS=11.35; ICV: $r=.72$, $p<.001$, TSS=145.92).

Cortical Development

PAE: Main effects and interactions with age. PAE's effects on thickness were minimal, with only the right-parahippocampal cortex being thicker (main effect), *p*=.017 (eTable 16), and the left-caudal-anterior-cingulate thinning more slowly in those with versus without PAE, *p*=.007 (eTable 18) (PAE×Age); neither passed FDR correction. Those with PAE (main effect) showed greater surface areas in 3 regions (left-inferior-temporal, right-middle-temporal, left-fusiform), *p*s≤.017, and smaller areas in 4 regions (right-postcentral, left-inferior-parietal, left-frontal-pole, left-medial-orbitofrontal), *p*s≤.050; none passed FDR correction (eTable 23). There were minimal PAE×Age interactions on surface area (left-entorhinal, left-interior-temporal, left-lateral-orbitofrontal, right-pericalcarine, *p*s≤.031), none passing FDR correction (eTable 25).

PTE: Main effects. Those with PTE had thinner cortices in 8 regions (bilateral-parahippocampal, bilateral-precentral, bilateral-paracentral, left-lateral-orbitofrontal, left-temporal-pole), *p*s≤.043; the bilateral-parahippocampal and left-lateral-orbitofrontal cortical effects passed FDR correction (eTable 17). Those with PTE showed thicker cortices in 5 regions (bilateral-pars-opercularis, right-transverse-temporal, right-pars-triangularis, right-caudal-anterior cingulate), none passing FDR correction, *p*s≤.045 (eTable 17) (Figure 2A). PTE was associated with reduced surface area in 6 regions (bilateral-precentral, right-posterior-cingulate, left-pericalcarine, right-entorhinal, left-postcentral) and increased area in 5 regions (bilateral-pars-triangularis, bilateral-insula, left-superior-frontal), *p*s≤.040, which did not pass FDR correction (eTable 24) (Figure 2B).

PTE: Interactions with age. There was a robust pattern of PTE being associated with steeper rates of cortical thinning, with there being FDR-corrected PTE×Age interactions in 11 frontal regions (bilateral-rostral-middle-frontal, bilateral-superior-frontal, bilateral-medial-

© 2025 Marshall AT et al. *JAMA Network Open*.

orbitofrontal, bilateral-rostral-anterior-cingulate, right-pars-orbitalis, left-frontal-pole, right-pars-triangularis) and 2 temporal regions (right-banks-of-the-superior-temporal-sulcus, left-inferior-temporal), *p*s≤.005; 9 additional regions (including 3 frontal and 4 temporal regions) showed non-FDR-corrected PTE×Age interactions, *p*s≤.043 (eTable 19) (Figure 3). There were PTE×Age interactions on surface area in 5 regions (left-supramarginal, right-lateral-orbitofrontal, left-frontal-pole, left-paracentral, left-pericalcarine), *p*s≤.038, but they did not pass FDR correction (eTable 26).

PAE×PTE interactions. There were no FDR-corrected PAE×PTE or PAE×PTE×Age interactions on thickness (eTables 20-21) or surface area (eTables 27-28).

© 2025 Marshall AT et al. *JAMA Network Open*.

Post Hoc Analyses

Knowing-of-Pregnancy PTE. These analyses included participants of caregivers reporting PTE after learning of the pregnancy (*n*=262) and the same 4,678 non-PTE participants from the initial analyses; here, between 10 and 214 participants were removed from individual-ROI analyses due to outlying/missing data. Model equations were identical to the initial analyses above. Collapsed across timepoints, PTE was associated with thinner bilateral-parahippocampal cortices, *p*s≤.038, and thicker bilateral-lingual cortices, *p*s≤.030, but these effects did not pass FDR correction (eTable 45). There were PTE×Age interactions in 5 regions (left-frontal-pole, right-inferior-parietal, right-pars-orbitalis, left-superior-frontal, left-rostral-anterior-cingulate), *p*s≤.039, none passing FDR correction (eTable 47). There were no FDR-corrected PAE×PTE or PAE×PTE×Age interactions (eTables 48-49).

PTE without PAE. These analyses included 3,917 participants whose caregivers did not report alcohol use during pregnancy (PTE: *n*=366, regardless of before/after learning of pregnancy; non-PTE: *n*=3,551); between 9 and 163 participants were removed from individual-ROI analyses due to outlying/missing data. Model equations were identical to the initial analyses except PAE-included factors were removed. Across timepoints, PTE was associated with thinner cortices in 4 regions (bilateral-parahippocampal, right-precentral, left-lateral-orbitofrontal), *p*s≤.048, with only the effect on right-parahippocampal thickness passing FDR correction; in 6 regions, PTE was associated with thicker cortices (bilateral-cuneus, right transverse-temporal, left-pars-opercularis, right-superior-temporal, right-temporal-pole), *p*s≤.039, which did not pass FDR correction (eTable 51). There were PTE×Age interactions in 9 regions (bilateral-inferior-temporal, bilateral-rostral-anterior-cingulate, bilateral-rostral-middle-frontal, left-frontal-pole,

© 2025 Marshall AT et al. *JAMA Network Open*.

right-medial-orbitofrontal, left-superior-frontal), *p*s≤.036; only the association of faster cortical thinning of the left-frontal-pole in the PTE group passed FDR correction (eTable 52).

© 2025 Marshall AT et al. *JAMA Network Open*.

Exploratory Analyses: Developmental Outcomes

There were significant, FDR-corrected PTE×Age on interactions on cortical thickness (CT) in 13 regions. Accordingly, we calculated individuals' APC_{CT} in each of these regions, so as to provide an individual-differences cortical-change measure to correlate with developmental-outcome measures. eFigure 6 shows these APC_{CT} distributions for both the PTE and Non-PTE groups. Of 5,417 participants (PTE: 739; Non-PTE: n = 4,678), the following number of participants (per region) were removed due to their APC_{CT} data being outliers or missing: right-banks-of-the-superior-temporal-sulcus (PTE: n = 16; Non-PTE: n = 93), left-frontal-pole (PTE: n = 45; Non-PTE: n = 226), left-inferior-temporal (PTE: n = 48; Non-PTE: n = 221), left-medial-orbital-frontal (PTE: n = 14; Non-PTE: n = 85), right-medial-orbital-frontal (PTE: n = 17; Non-PTE: n = 107), right-pars-orbitalis (PTE: n = 23; Non-PTE: 134), right-pars-triangularis (PTE: n = 37; Non-PTE: n = 165), left-rostral-anterior-cingulate (PTE: n = 20; Non-PTE: n = 113), right-rostral-anterior-cingulate (PTE: n = 18; Non-PTE = 125), left-rostral-middle-frontal (PTE: n = 40; Non-PTE: n = 244), right-rostral-middle-frontal (PTE: n = 38; Non-PTE: n = 207), left-superior-frontal (PTE: n = 53; Non-PTE: n = 243), and right-superior-frontal (PTE: n = 40; Non-PTE: n = 226). Thus, ≥92.8% and ≥94.8% of PTE and Non-PTE APC_{CT} data were maintained in analysis.

Except for the right-pars-triangularis cortex (t = 1.88, p = .061), APC_{CT} was significantly greater in the Non-PTE group than in the PTE group, reflecting faster rates of cortical thinning given PTE (in accordance with the linear mixed-effects analyses): right-banks-of-the-superior-temporal-sulcus (t = 3.10, p = .002), left-frontal-pole (t = 2.14, p = .033), left-inferior-temporal (t = 3.14, p = .002), left-medial-orbital-frontal (t = 2.19, p = .029), right-medial-orbital-frontal (t = 2.56, p = .011), right-pars-orbitalis (t = 2.65, p = .008), left-rostral-anterior-cingulate (t = 2.20, p

= .028), right-rostral-anterior-cingulate (t = 2.90, p = .004), left-rostral-middle-frontal (t = 3.46, p = .001), right-rostral-middle-frontal (t = 3.18, p = .001), left-superior-frontal (t = 2.89, p = .004), and right-superior-frontal (t = 3.17, p = .002).

Next, we regressed the developmental-outcome data on APC_{CT} and determined whether PTE significantly moderated associations between APC_{CT} and the developmental-outcome data (PTE×APC_{CT}). For each outcome-by-region analysis, participants' developmental-outcome data were considered outliers (and excluded) if they exceeded more than three scaled median absolute deviations away from the median.[15] eTables 53-60 show the number of participants within each analysis. All analyses included at least 4,340 individuals (i.e., ≥80.1% of the sample in the initial set of cortical-development analyses).

Figure 4A shows heatmaps of the strengths (i.e., effect sizes, r_p) of these associations and interactions. Of the 390 models (13 regions × 30 outcomes), there were significant 26 significant associations between APC_{CT} and neurodevelopmental outcomes (BIS-BAS: 16 associations; CBCL: 2 associations; SDSC: 1 association; UPPS: 7 associations); there were 19 significant PTE×APC_{CT} interactions (BIS-BAS: 4 interactions; CBCL: 4 interactions; SDSC: 1 interaction; UPPS: 10 interactions). Note that greater values of APC_{CT} reflect slower cortical thinning (or faster cortical thickening) and lower values of APC_{CT} reflect faster cortical thinning (or slower cortical thickening); in other words, *positive* associations indicate that greater values of the developmental outcomes (or greater difference scores) were associated with *slower cortical thinning* (or faster cortical thickening), and *negative* associations indicate that greater values of these developmental outcomes (or greater difference scores) were associated with *faster cortical thinning* (or slower cortical thickening). Statistical output for these models is provided separately for each scale: BIS-BAS (eTables 53-54), CBCL (eTables 55-56), SDSC (eTables 57-58), and

© 2025 Marshall AT et al. *JAMA Network Open*.

UPPS (eTables 59-60). The four region-outcome pairs with both significant associations of APC_{CT} and PTE×APC_{CT} interactions (right-banks-of-the-superior-temporal-sulcus – negative urgency at the two-year appointment; left-frontal-pole – BAS-fun seeking at the two-year appointment; right-pars-orbitalis – total sleep disorders at the two-year appointment; right-pars-orbitalis – CBCL externalizing behavior at the two-year appointment) are described in the main text (see Figure 4B).

© 2025 Marshall AT et al. *JAMA Network Open*.

eReferences.

1. Talati A, Wickramaratne PJ, Wesselhoeft R, Weissman MM. Prenatal tobacco exposure, birthweight, and offspring psychopathology. *Psychiatry Research*. 2017;252:346-352. doi:10.1016/j.psychres.2017.03.016

2. El Marroun H, Schmidt MN, Franken IHA, et al. Prenatal tobacco exposure and brain morphology: A prospective study in young children. *Neuropsychopharmacology*. 2014;39:792-800. doi:10.1038/npp.2013.273

3. de Kieviet JF, Zoetebier L, van Elburg RM, Vermeulen RJ, Oosterlaan J. Brain development of very preterm and very low-birthweight children in childhood and adolescence: A meta-analysis. *Developmental Medicine & Child Neurology*. 2012;54(4):313-323. doi:10.1111/j.1469-8749.2011.04216.x

4. Barch DM, Albaugh MD, Avenevoli S, et al. Demographic, physical and mental health assessments in the adolescent brain and cognitive development study: Rationale and description. *Developmental Cognitive Neuroscience*. 2018;32:55-66. doi:10.1016/j.dcn.2017.10.010

5. Barch DM, Albaugh MD, Baskin-Sommers A, et al. Demographic and mental health assessments in the adolescent brain and cognitive development study: Updates and age-related trajectories. *Developmental Cognitive Neuroscience*. 2021;52:101031. doi:10.1016/j.dcn.2021.101031

6. Carver CS, White TL. Behavioral inhibition, behavioral activation, and affective responses to impending reward and punishment: The BIS/BAS scales. *Journal of Personality and Social Psychology*. 1994;67(2):319-333.

7. Pagliaccio D, Luking KR, Anokhin AP, et al. Revising the BIS/BAS Scale to study development: Measurement invariance and normative effects of age and sex from childhood through adulthood. *Psychological Assessment*. 2016;28(4):429-442. doi:10.1037/pas0000186

8. Achenbach TM. *The Achenbach system of empirically based assessment (ASEBA): Development, findings, theory, and applications*. University of Vermont, Research Center for Children, Youth, & Families; 2009.

9. Bruni O, Ottaviano S, Guidetti V, et al. The Sleep Disturbance Scale for Children (SDSC). Construction and validation of an instrument to evaluate sleep disturbances in childhood and adolescence. *Journal of Sleep Research*. 1996;5(4):251-261. doi:10.1111/j.1365-2869.1996.00251.x

10. Nakagawa S, Cuthill IC. Effect size, confidence interval and statistical significance: A practical guide for biologists. *Biological Reviews*. 2007;82:591-605. doi:10.1111/j.1469-185X.2007.00027.x

11. Aloe AM, Thompson CG. The synthesis of partial effect sizes. *journal of the Society for Social Work and Research*. 2013;4(4):390-405. doi:10.5243/jsswr.2013.24

12. Treit S, Zhou D, Chudley AE, et al. Relationships between head circumference, brain volume and cognition in children with prenatal alcohol exposure. *PLOS ONE*. 2016;11(2):e0150370. doi:10.1371/journal.pone.0150370

13. Marshall AT, Adise S, Kan EC, Sowell ER. Longitudinal sex-at-birth and age analyses of cortical structure in the ABCD Study®. *bioRxiv*. 2024;doi:10.1101/2024.06.10.598367

14. Mills KL, Siegmund KD, Tamnes CK, et al. Inter-individual variability in structural brain development from late childhood to young adulthood. *NeuroImage*. 2021;242:118450. doi:10.1016/j.neuroimage.2021.118450

© 2025 Marshall AT et al. *JAMA Network Open*.

15. Leys C, Ley C, Klein O, Bernard P, Licata L. Detecting outliers: Do not use standard deviation around the mean, use absolute deviation around the median. *Journal of Experimental Social Psychology*. 2013;49(4):764-766. doi:10.1016/j.jesp.2013.03.013

© 2025 Marshall AT et al. *JAMA Network Open*.

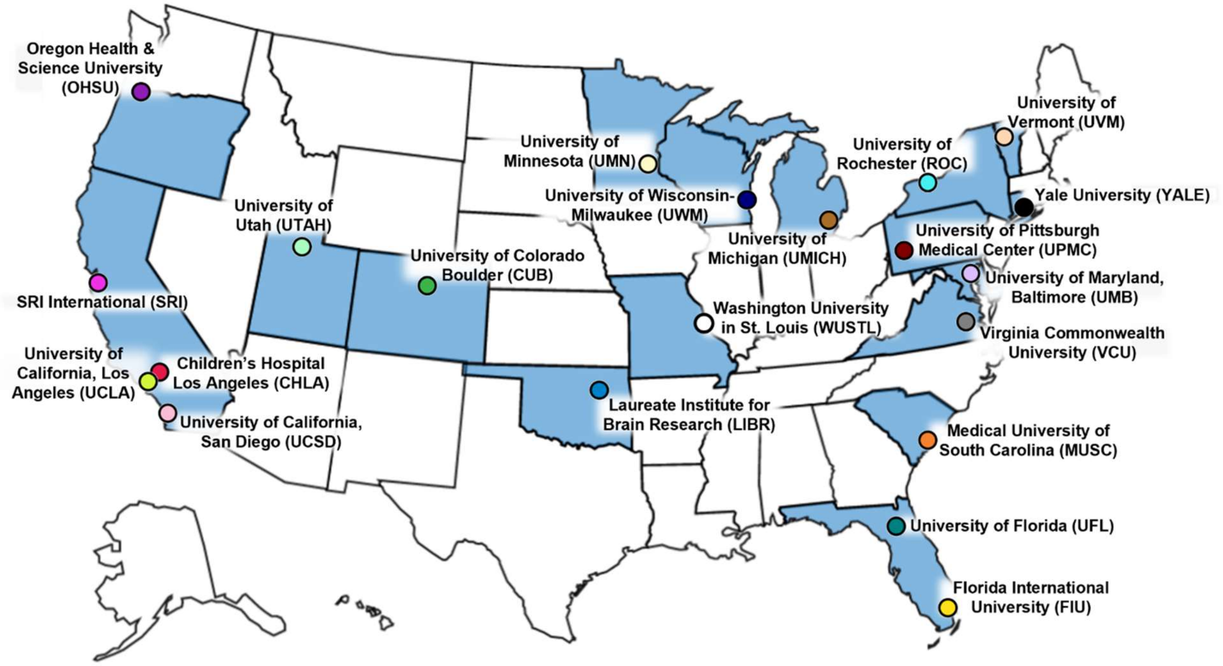

eFigure 1. US Map of the Locations of the Adolescent Brain Cognitive Development (ABCD) Study

Each state with an ABCD data-collection site is shaded, and the full name and abbreviation of that site accompanies a circular marker of the approximate location of that site within the state.

© 2025 Marshall AT et al. *JAMA Network Open*.

Inclusion/Exclusion Rule	Participants Remaining
Include participants with Adolescent Brain Cognitive Development (ABCD) Data Release 4.0	11,876
Include participants with baseline or two-year data	11,876
Exclude participants recommended for exclusion per ABCD's release notes	11,868
Include participants whose T_1-weighted neuroimaging data passed quality control	11,659
Include participants with baseline and two-year structural magnetic resonance imaging data	7,432
Exclude participants with missing or outlying birthweight data	6,696
Include singletons or one sibling per family*	5,713
Exclude participants whose caregivers were unaware of the pregnant person's alcohol/tobacco use after learning/knowing of the pregnancy	5,417

eTable 61. Inclusionary and Exclusionary Criteria for Analysis

*Because ABCD includes siblings, we controlled for family relatedness by only including singletons or one sibling per family; we prioritized siblings based on prenatal alcohol exposure (PAE)/prenatal tobacco exposure (PTE) and data completeness (yes/no responses to PTE/PAE before/after learning of the pregnancy: PTE/Before, PTE/After, PAE/Before, PAE/After). If siblings had identical sets of yes/no/don't know (DK) responses, we randomly selected one sibling from the corresponding family groups using MATLAB's *datasample* function (seed=1) [MATLAB Version: 9.13.0.2126072 (R2022b) Update 3]. Otherwise, we included the sibling based on (1) more "yes" responses to PTE/After and PAE/After pregnancy recognition, (2) more "yes" responses total, and (3) fewest DK responses.

© 2025 Marshall AT et al. *JAMA Network Open*.

	ABCD Cohort – Baseline (%)[a]	Sample in This Report - Baseline (%)[a]	Cohort vs. Sample
**Youth Sex-Assigned-at-Birth**			$\chi(1)^2 = 5.45$, $p = .020$
Male	6,196 (52.2)	2,912 (53.8)	
Female	5,680 (47.8)	2,505 (46.2)	
**Annual Household Income**			$\chi(3)^2 = 22.42$, $p < .001$
<$50K (Low)	3,223 (27.1)	1,381 (25.5)	
$50-100K (Mid)	3,071 (25.9)	1,509 (27.9)	
>$100K (High)	4,564 (38.4)	2,123 (39.2)	
Missing/Undefined	1,018 (8.6)	404 (7.5)	
**Caregiver Education**			$\chi(5)^2 = 18.53$, $p = .002$
≤12th Grade/No Diploma	593 (5.0)	223 (4.1)	
High-school Graduate/GED or Equivalent	1,132 (9.5)	456 (8.4)	
Some College, No Degree/Associate's Degree	3,079 (25.9)	1,416 (26.1)	
Bachelor's Degree	3,015 (25.4)	1,427 (26.3)	
Master's/Professional Degree, Doctorate	4,043 (34.0)	1,888 (34.9)	
Missing/Undefined	NR (NR)	NR (NR)	
**Youth Race**			$\chi(6)^2 = 44.97$, $p < .001$
American Indian / Alaska Native	62 (0.5)	24 (0.4)	
Asian	275 (2.3)	95 (1.8)	
Black	1,869 (15.7)	724 (13.4)	
Native Hawaiian / Pacific Islander	NR (NR)	NR (NR)	
Other	1,959 (16.5)	867 (16.0)	
White	7,524 (63.4)	3,640 (67.2)	
Missing/Undefined	171 (1.4)	60 (1.1)	
**Youth Ethnicity**			$\chi(2)^2 = 6.61$, $p = .037$
Hispanic	2,411 (20.3)	1,048 (19.3)	
Not Hispanic	9,312 (78.4)	4,314 (79.6)	
Missing/Undefined	153 (1.3)	55 (1.0)	
**Total**	11,876 (100)	5,417 (100)	

eTable 62. Participant Demographics

The data in the "ABCD Cohort" column refer to those derived from the 4.0 ABCD Study Data Release (October 2021). The "Other" Race/Ethnicity category includes those who identified "Other Race" and those who identified more than one race (i.e., multiracial). The third column shows the chi-squared (χ^2) statistics (with the degrees of freedom in parentheses) comparing the observed number of individuals in the sample (per level of each demographic variable) ("Sample in This Report") versus the expected number, given proportions at each level of the larger cohort ("ABCD Cohort"). p = p-value. [a] Values of small cell sizes ($n < 20$) (and the corresponding %) were suppressed (i.e., noted by NR), but those values were used to compute the χ^2 statistics. See

© 2025 Marshall AT et al. *JAMA Network Open*.

Supplemental eTables 63-64 for the demographics of the PAE versus Non-PAE groups and PTE versus Non-PTE groups.

© 2025 Marshall AT et al. *JAMA Network Open*.

	PAE (%)[a]	Non-PAE (%)[a]	PAE vs. Non-PAE
**Youth Sex-Assigned-at-Birth**			$\chi(1)^2 = 4.43$, $p = .035$
Male	777 (51.8)	2,135 (54.5)	
Female	723 (48.2)	1,782 (45.5)	
**Annual Household Income**			$\chi(3)^2 = 159.40$, $p < .001$
<$50K (Low)	264 (17.6)	1,117 (28.5)	
$50-100K (Mid)	440 (29.3)	1,069 (27.3)	
>$100K (High)	726 (48.4)	1,397 (35.7)	
Missing/Undefined	70 (4.7)	334 (8.5)	
**Caregiver Education**			$\chi(5)^2 = 129.55$, $p < .001$
≤12th Grade/No Diploma	NR (NR)	206 (5.3)	
High-school Graduate/GED or Equivalent	78 (5.2)	378 (9.7)	
Some College, No Degree/Associate's Degree	357 (23.8)	1,059 (27.0)	
Bachelor's Degree	430 (28.7)	997 (25.5)	
Master's/Professional Degree, Doctorate	618 (41.2)	1,270 (32.4)	
Missing/Undefined	NR (NR)	NR (NR)	
**Youth Race**			$\chi(6)^2 = 105.30$, $p < .001$
American Indian / Alaska Native	NR (NR)	NR (NR)	
Asian	NR (NR)	80 (2.0)	
Black	114 (7.6)	610 (15.6)	
Native Hawaiian / Pacific Islander	NR (NR)	NR (NR)	
Other	236 (15.7)	631 (16.1)	
White	1,112 (74.8)	2,518 (64.3)	
Missing/Undefined	NR (NR)	53 (1.4)	
**Youth Ethnicity**			$\chi(2)^2 = 44.10$, $p < .001$
Hispanic	216 (14.4)	832 (21.2)	
Not Hispanic	1,272 (84.8)	3,042 (77.7)	
Missing/Undefined	NR (NR)	43 (1.1)	
**Total**	1,500 (100%)	3,917 (100%)	

eTable 63. Demographics of Youth Participants in This Study Reported by Caregivers as Being Prenatally Exposed and Not Exposed to Alcohol (PAE and Non-PAE)
The third column shows the chi-squared (χ^2) statistics (with the degrees of freedom in parentheses) comparing the proportion of individuals in the PAE group (per level of each demographic variable) relative to the corresponding proportions of individuals in the Non-PAE group. p = p-value. [a]Values of small cell sizes ($n < 20$) (and the corresponding %) were suppressed (i.e., noted by NR), but those values were used to compute the χ^2 statistics.

© 2025 Marshall AT et al. *JAMA Network Open*.

	PTE (%)[a]	Non-PTE (%)[a]	PTE vs. Non-PTE
**Youth Sex-Assigned-at-Birth**			$\chi(1)^2 = 0.77$, $p = .381$
Male	387 (52.4)	2,525 (54.0)	
Female	352 (47.6)	2,153 (46.0)	
**Annual Household Income**			$\chi(3)^2 = 311.07$, $p < .001$
<$50K (Low)	331 (44.8)	1,050 (22.4)	
$50-100K (Mid)	208 (28.1)	1,301 (27.8)	
>$100K (High)	117 (15.8)	2,006 (42.9)	
Missing/Undefined	83 (11.2)	321 (6.9)	
**Caregiver Education**			$\chi(5)^2 = 526.68$, $p < .001$
≤12th Grade/No Diploma	43 (5.8)	180 (3.8)	
High-school Graduate/GED or Equivalent	131 (17.7)	325 (6.9)	
Some College, No Degree/Associate's Degree	358 (48.4)	1,058 (22.6)	
Bachelor's Degree	119 (16.1)	1,308 (28.0)	
Master's/Professional Degree, Doctorate	86 (11.6)	1,802 (38.5)	
Missing/Undefined	NR (NR)	NR (NR)	
**Youth Race**			$\chi(6)^2 = 58.36$, $p < .001$
American Indian / Alaska Native	NR (NR)	NR (NR)	
Asian	NR (NR)	91 (1.9)	
Black	136 (18.4)	588 (12.6)	
Native Hawaiian / Pacific Islander	NR (NR)	NR (NR)	
Other	156 (21.1)	711 (15.2)	
White	428 (57.9)	3,212 (68.7)	
Missing/Undefined	NR (NR)	51 (1.1)	
**Youth Ethnicity**			$\chi(2)^2 = 4.12$, $p = .127$
Hispanic	130 (17.6)	918 (19.6)	
Not Hispanic	598 (80.9)	3,716 (79.4)	
Missing/Undefined	NR (NR)	44 (0.9)	
**Total**	739 (100%)	4,678 (100%)	

eTable 64. Demographics of Youth Participants in This Study Reported by Caregivers as Being Prenatally Exposed and Not Exposed to Tobacco (PTE and Non-PTE)

The third column shows the chi-squared (χ^2) statistics (with the degrees of freedom in parentheses) comparing the proportion of individuals in the PTE group (per level of each demographic variable) relative to the corresponding proportions of individuals in the Non-PTE group. p = p-value. [a]Values of small cell sizes ($n < 20$) (and the corresponding %) were suppressed (i.e., noted by NR), but those values were used to compute the χ^2 statistics.

© 2025 Marshall AT et al. *JAMA Network Open*.

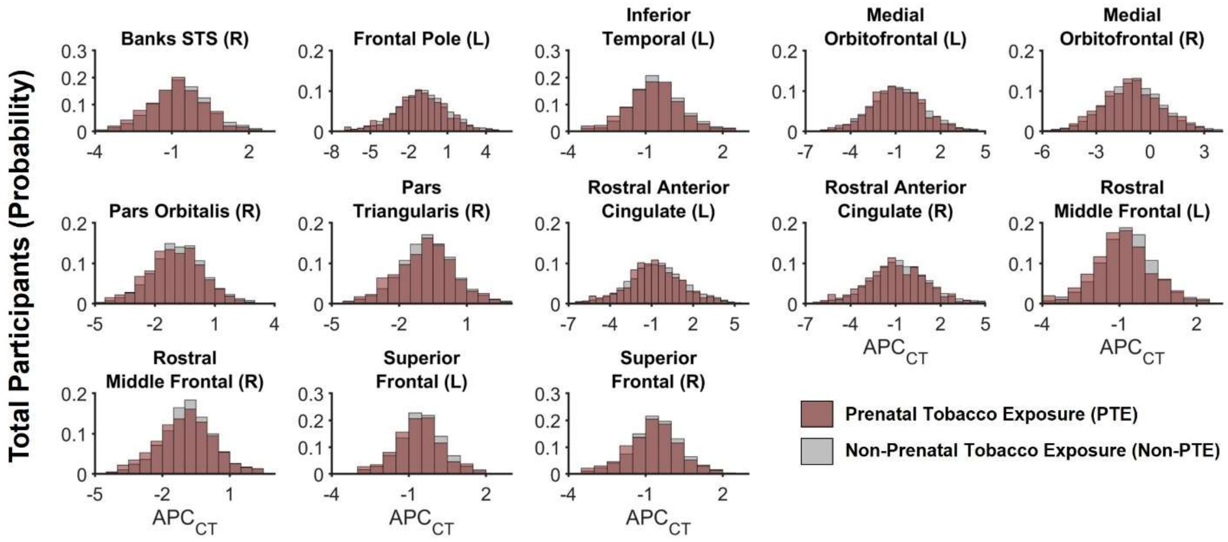

eFigure 2. Annual Percentage Change (APC) for Cortical Thickness

For the regions exhibiting significant Prenatal Tobacco Exposure (PTE) × Age interactions on cortical thickness (Figure 3), probability-density histograms of individual differences in cortical-thickness (CT) development (i.e., annual percentage change, APC_{CT}) for those with and without PTE. Note that greater values of APC_{CT} reflect slower cortical thinning (or faster cortical thickening) and lower values of APC_{CT} reflect faster cortical thinning (or slower cortical thickening). Banks STS = banks of the superior temporal sulcus. L = left hemisphere. R = right hemisphere.

© 2025 Marshall AT et al. *JAMA Network Open*.
