# Supplementary material for: Prenatal Tobacco and Alcohol Exposure and Cortical Change Among Youths
Source: JAMA Netw Open. 2025 Jun 20;8(6):e2516729. doi: 10.1001/jamanetworkopen.2025.16729 (PMC12181798; doi:10.1001/jamanetworkopen.2025.16729)
Supplement: Supplement 3. — Data Sharing Statement [file jamanetwopen-e2516729-s003.pdf]

Data Sharing Statement

Marshall. Cortical Change Among Youths and Prenatal Tobacco and Alcohol Exposure. *JAMA Netw Open*. Published June 20, 2025. doi:10.1001/jamanetworkopen.2025.16729

Data
Data available: Yes
Data types: Deidentified participant data, Data dictionary
How to access data: Data for the Adolescent Brain Cognitive Development (ABCD) Study are available via the NIH Brain Development Cohorts (NBDC) Biorepository Portal (https://nbdc.nida.nih.gov/) upon approved access to the data.
When available: beginning date: 06-01-2025

Supporting Documents
Document types: Statistical/analytic code
How to access documents: Statistical and analytical code can be made available by contacting Dr. Andrew Marshall: amarshall@chla.usc.edu
When available: With publication

Additional Information
Who can access the data: Anyone requesting the statistical/analytical code.
Types of analyses: For any purpose.
Mechanisms of data availability: With investigator support.
Any additional restrictions: The statistical/analytical code was written in MATLAB.
